# Short‐term and intermediate‐term performance and safety of left bundle branch pacing

**DOI:** 10.1111/jce.14463

**Published:** 2020-04-08

**Authors:** Jincun Guo, Linlin Li, Fanqi Meng, Maolong Su, Xinyi Huang, Simei Chen, Qiang Li, Dong Chang, Binni Cai

**Affiliations:** ^1^ Division of Cardiology Xiamen Cardiovascular Hospital, Xiamen University Xiamen Fujian China; ^2^ Division of Echocardiography Xiamen Cardiovascular Hospital, Xiamen University Xiamen Fujian China; ^3^ Division of Cardiac Function Xiamen Cardiovascular Hospital, Xiamen University Xiamen Fujian China

**Keywords:** feasibility, intermediate‐term, left bundle branch pacing, left bundle branch potential, safety

## Abstract

**Introduction:**

Left bundle branch pacing (LBBP) is a promising new method for patients with pacing indications. This study aims to evaluate the safety and feasibility of LBBP in a relatively longer time span.

**Methods and Results:**

A total of 164 patients were recruited for LBBP in this study. Among these patients, 148 patients had pacing indications due to symptomatic bradycardia while the other 16 patients had indications for cardiac resynchronization therapy (CRT). LBBP was successful in 89.0% (146/164) of all recruited patients. Intracardiac and surface electrographic parameters and image data were documented during the LBBP procedure. The mean paced QRS duration (pQRSD) and the mean stimulus to left ventricular activation time (stim‐LVAT) was 106.0 ± 12.9 ms and 64.4 ± 13.7 ms respectively. Left bundle branch (LBB) potentials were recorded in 89 patients. Forty‐three of whom had sick sinus syndrome (SSS), and 46 had atrioventricular block (AVB). The presence of LBB potential was more common in patients with SSS (82.7% vs 57.5%, *P* = .002). No significant differences in pQRSD, stim‐LVAT, or capture threshold were detected between patient groups with or without LBB potential. Patients were followed up at 1 month, 3 months, 6 months, and 1 year after the procedure. Pacing parameters and the echocardiographic data remained stable within a mean follow‐up period of 8.6 ± 4.3 months. No serious complication caused by this procedure was found in this study.

**Conclusions:**

Successful LBBP carried an aspect of short pQRSD and stim‐LVAT while the LBB potential was not the prerequisite and necessary feature. The LBBP procedure had a high success rate with satisfied and stable lead parameters during short and intermediate‐term observations.

## 1 INTRODUCTION

The adverse effects of right ventricular apex (RVA) pacing have long been realized, including electrical and mechanical asynchrony that will increase the risk of atrial fibrillation, heart failure, and bring a higher mortality rate.^1^, ^2^ Initial investigations have confirmed the feasibility and safety of left bundle branch pacing (LBBP) via short‐term and medium‐term follow‐ups in relatively small cohorts.^3^, ^4^, ^5^, ^6^ As LBBP is an innovative technique, there is only limited information about its outcomes in a relatively longer term. In this report, we described the short‐term and intermediate‐term performance and safety of LBBP in a cohort of Chinese patients with bradycardia‐related pacing indications or resynchronization indications in our center.

## 2 METHODS

### 2.1 Participants recruitment

Consecutive patients with pacing indications including bradycardia and heart failure combined with complete left bundle branch block (CLBBB) in Xiamen Cardiovascular Hospital, Xiamen University from January 2018 to September 2019 were recruited for LBBP attempts in the present study. The approval of the Ethics Committee of Xiamen Cardiovascular Hospital, Xiamen University was obtained before patient enrollment, and informed consent was obtained from all recruited patients. The trial was conducted in accordance with the principles of the Declaration of Helsinki and the guidelines for Good Clinical Practice (EMA/CPMP/CIH/135/1995).

### 2.2 Lead implantation

LBBP was achieved by a transventricular septal method in the basal ventricular septum as described elsewhere.^7^ Briefly, the Select Secure (model 3830, 69 cm, Medtronic Inc, Minneapolis, MN) pacing lead was introduced through a fixed curve sheath (C315 HIS, Medtronic Inc) toward the right ventricle beyond the tricuspid annulus. During the procedure, the His bundle region was identified as an anatomic marker before further advancing the lead toward the cardiac apex by 1.0 to 2.0 cm. Once the paced QRS morphology showed a “W” pattern in V1, the lead was perpendicularly screwed in. When screwing, the unipolar electrode pacing pattern and impedance were consecutively monitored. The lead was finally fixed when the paced QRS morphology showed a “QR/Qr” pattern in V1, and the stimulus to left ventricular activation time (stim‐LVAT) was the shortest and consistent during high and low outputs in V5 or V6.^8^ The right atrial lead was implanted in the right atrial appendage and connected to the atrial port. The LBBP lead was connected to the ventricular port if a dual‐chamber pacemaker was implanted. For patients who failed in LBBP, right ventricular septal pacing (RVSP) was performed as an alternative. The LV leads were implanted in the lateral or postlateral left ventricular vein if possible as mentioned elsewhere.^9^ In patients with normal sinus rhythm undergoing CRT‐pacemakers(P), the LBBP lead was connected to the right ventricular (RV) port and the left ventricular (LV) lead to the LV port. In patients with normal sinus rhythm undergoing CRT‐defibrillators(D), the LBBP lead was connected to the LV port and an implantable cardioverter‐defibrillator lead was implanted in the RVA and connected to RV port.

### 2.3 Electrocardiographic measurements

Twelve‐lead surface electrocardiography (ECG) was recorded by the GE CardioLab Electrophysiology recording system (GE Healthcare Inc, Marlborough, MA) at 100 mm/s. The intracardiac electrogram (IEGM) was recorded from the tip electrode of 3830 lead during lead implantation. LBB potential was defined as an isolated signal appeared in advance of the QRS complex in intrinsic rhythm when the tip electrode reached the position right beneath the endocardium of left ventricle. The intrinsic QRS duration (QRSD), paced QRS duration (pQRSD), stim‐LVAT, and potential to ventricular interval (PVI) were measured in sequence. First, the QRSD was measured in the 12‐lead ECG taken during implantation, and the duration was measured from the first to the last sharp vector of QRS complexes crossing the isoelectric line in 12 leads to the last deflection of the complex. Second, the pQRSD was measured from the onset of the first deviation from baseline for selective LBBP and from the onset of steepest deflection in nonselective LBBP to the end of the last deflection of the QRS complex in 12 leads. The selective LBBP was defined as capturing the LBB with a discrete component between the stimulus and onset of QRS complex under threshold output, while the nonselective LBBP captured both the LBB and the local myocardium and no discrete interval presented between the pacing spike and surface ECG QRS onset. Following that, the stim‐LVAT was measured from the pacing stimulus to the peak of R‐wave in lead V5 or V6. At last, the PVI was assessed from the LBB potential to the onset of QRS complex.

### 2.4 Data collection and follow‐up

Baseline characteristics of participants were collected at enrollment. During implantation, the intracardiac, surface electrographic parameters, and imaging data were documented. Lead parameters, ECG morphology, and echocardiographic data, including left ventricular ejection fraction (LVEF), left ventricular end‐diastolic internal dimension (LVIDd), and interventricular septal thickness (IVSd) were recorded at least 12 to 24 hours before the procedure and each follow‐up visit. Patients were followed up at 3 days after the operation, 1 month, 3 months, 6 months, and 1 year after implantation. Possible complications such as infections, pericardial effusion, capture threshold elevation, lead dislodgment, and lead deficiencies were routinely tracked. Transient ischemic attacks or stroke‐like symptoms were also recorded if there was any. "Chronic capture threshold elevation" was defined as a situation when the threshold was higher than 2.5 V@ 0.4 millisecond or was more than 1 V higher than the threshold instantly after implantation. Location and depth of the lead within the interventricular septum and severity of the tricuspid valve regurgitation (TVR) were assessed at each follow‐up visit as well.

### 2.5 Statistical analysis

Continuous and categorical variables were expressed as the mean ± SD and percentages, respectively. Differences between two groups were compared using the Student *t* test for continuous variables, and the *χ* ^2^ test was used for categorical data. A value of *P* < .05 was considered statistically significant. All statistical analyses were performed using SPSS Statistics version 22.0 (Chicago, IL).

## 3 RESULTS

### 3.1 Baseline characteristics

In total, 164 consecutive patients with bradycardia‐related pacing indications or CRT indications were recruited for LBBP attempts, and the LBBP success rate was 89.0% (146/164). Detailed baseline characteristics of the recruited patients were described in Table 1. In brief, 80 patients (54.8%) were male, and the mean age was 64.3 ± 10.8 years (ranges from 14 to 93 years). Sixteen patients had CRT indications with typical LBBB detected via EKG, 148 patients had bradycardia‐related pacing indications. Among whom, 93 (56.7%) patients had the pacing indication of atrioventricular block (AVB), and the other 55 (33.5%) patients had the indication of sinus node disease. In addition, in the 146 patients received LBBP, single‐chamber pacemakers were implanted in 15 (15/146, 10.3%) patients, dual‐chamber pacemakers in 117 (117/146, 80.1%) patients, and triple chamber pacemakers or defibrillators in the rest 14 (14/146, 9.6%) patients.

**Table 1:** Baseline and procedural characteristics

Total number of patients	164
Successful LBBP	146
Age (y)	64.3 ± 10.8
Men (%)	48.7%
Coronary artery disease	26
Atrial fibrillation	16
Hypertension	46
Cardiomyopathy	16
Indication of pacing	
Sinus node dysfunction	55
AVB	93
Cardiac resynchronization therapy	16
Baseline ECG characteristics	
QRS duration (ms)	107.6 ± 31.1
CLBBB	17
CRBBB	8
Type of pacemaker	
VVI	15
DDD	117
CRT	14
LBB potential (%)	89/164(54.3%)
LBB potential amplitude (mV)	0.3 ± 0.1
PVI (ms)	18.5 ± 5.6
Pacing‐QRS duration (ms)	106.0 ± 12.9
Stim‐LVAT (ms)	64.4 ± 13.7

Abbreviations: AVB, atrioventricular block; ECG, electrocardiogram; CLBBB, complete left bundle branch block; CRBBB, complete right bundle branch; CRT, cardiac resynchronization therapy; LBB, left bundle branch; LBBP, left bundle branch pacing; PVI, potential to ventricular interval; stim‐LVAT, stimulus to left ventricular activation time.

In this study, 11.0% (18/164) patients failed in LBBP implantation. Among whom, 16 patients with bradycardia received RVSP, and the other two patients with CRT indications received bi‐ventricular pacing (BiV). In addition, in 13 patients who failed in LBBP, difficulties in screwing the 3830 lead into the deep ventricular septum were recorded. Among whom five cases had local hypertrophy secondary to hypertension or aortic stenosis, two cases were complicated with DCM and significant septal myocardial fibrosis as confirmed by magnetic resonance imaging, and insufficient sheath support was witnessed in six cases due to significant right atrium enlargement and cardiac clockwise rotation. In another two patients with old septal myocardial infarction, unacceptable lead parameters were documented with either too high thresholds or too low R‐wave amplitude, and thus RVSP was preformed instead of LBBP. Loss of LBB capture occurred in three patients after withdrawal of the sheath during procedure. RVSP was subsequently conducted as an alternative method in lieu of LBBP.

Among this cohort, 47.2% (69/146) patients had a follow‐up period that equaled to or was longer than 12 months and 63.0% (92/146) patients had a follow‐up period that was longer than 6 months. The mean follow‐up duration was 8.6 ± 4.3 months (ranges from 3 to 18 months).

### 3.2 Electrophysiological characteristics

**Figure 1:**
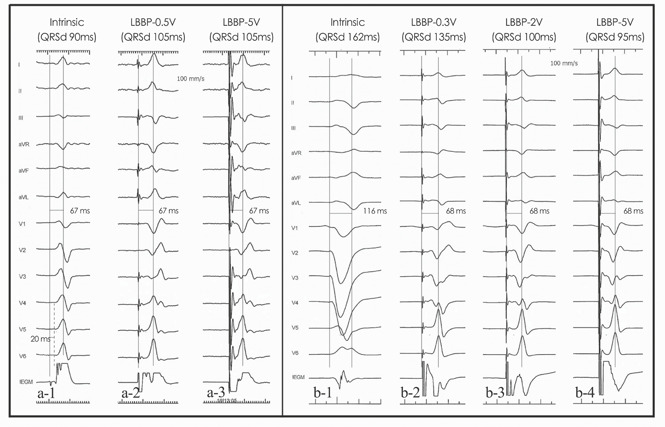
Electrophysiological characteristics of LBBP. Surface and intracardiac electrograms were taken from patients under LBBP. In the first patient (a), a LBB potential was recorded with the PVI being 20 ms, and the potential to LVAT duration was equal to 67 ms (a1). There was consistency among LVAT at low output (a2, 0.5 V@ 0.4 ms), high output (a3, 5 V@ 0.4 ms) and intrinsic activation. In another patient who underwent LBBP (b), the intrinsic QRS complex presented LBBB morphology, no LBB potential was recorded and the intrinsic LVAT was 116 ms (b1). The paced QRS wave presented “rSR” morphology in V1 with an isoelectric line before the onset of QRS wave which was deemed as selective LBB capture under capture threshold (0.3 V@ 0.4 ms, b2). As the output increased, the isoelectric line disappeared with the “R” wave of V1 decreased and the S wave of V5, V6 shallowed which was deemed as nonselective LBB capture (b3 and b4). The stim‐LVAT was 68 ms and consistent during high and low output which was shorter than intrinsic LVAT. LBB, left bundle branch; LBBB, left bundle branch block; LVAT, left ventricular activation time; PVI, potential to ventricular interval; stim‐LVAT, stimulus to left ventricular activation time

**Figure 2:**
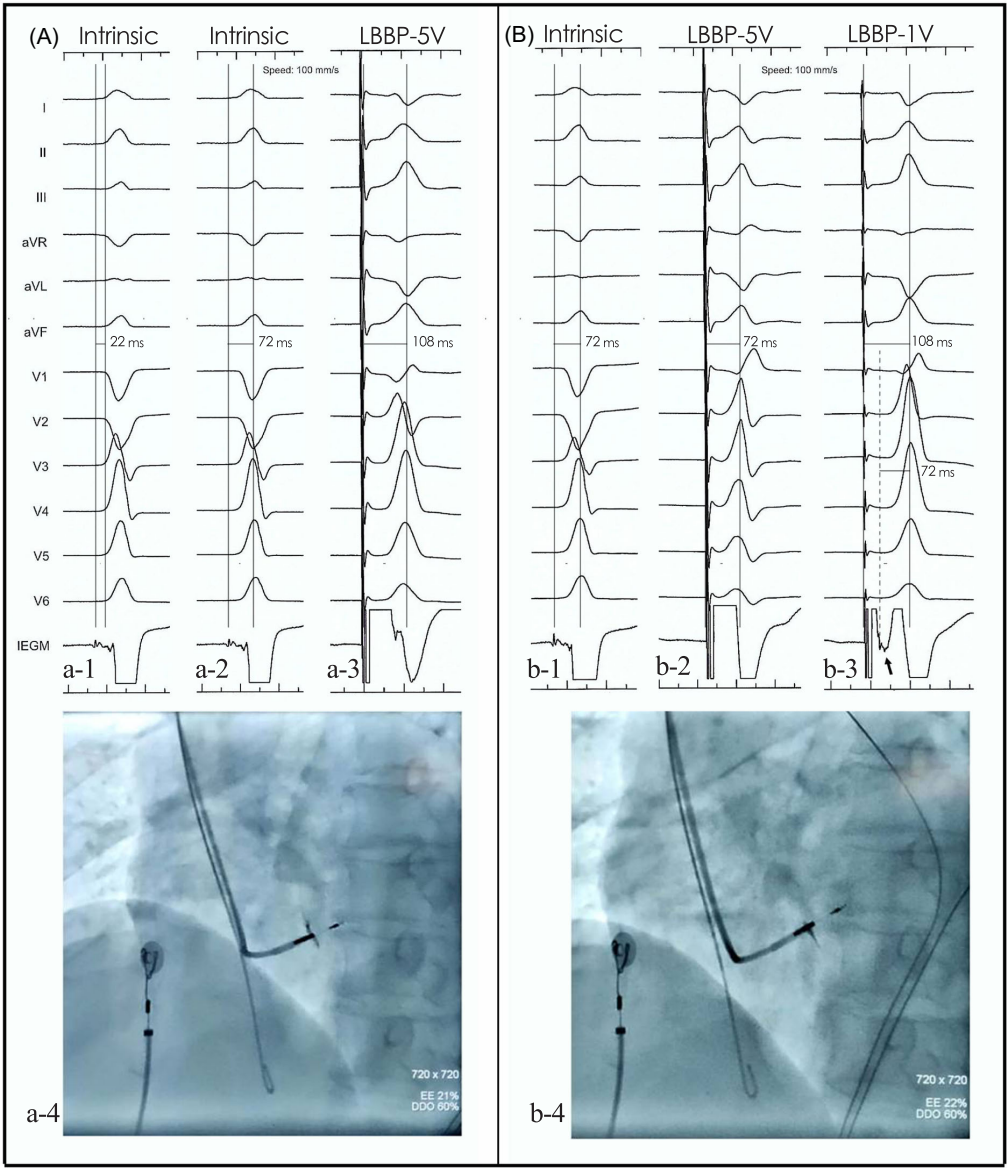
Dynamic changes of stim‐LVAT and potential amplitude during LBBP procedure. In one patient, a small LBB potential was recorded with a PVI of 22 ms (a1) and a LVAT of 72 ms under intrinsic conduction (a2). The paced QRS complex presented QR morphology and the stim‐LVAT was 108 ms under high output (5 V@ 0.4 ms, a3). The amplitude of LBB potential increased as the lead was slightly screwed in (b1). Under the high output, the R‐wave in V1 enlarged and a s wave presented in V5 and V6. In addition, there was a consistency between high output (5 V@ 0.4 ms) and intrinsic activation on LVAT (b2). An abrupt change of stim‐LVAT was convinced under low (1 V@ 0.4 ms) and high outputs (b2 and b3). A high‐frequency signal (black arrow) was recorded on IEGM under low output, and the duration from the signal to the peak of R‐wave in lead V5 was consistent with LVAT under high output or intrinsic conduction (b3). The lead depth in the septum was showed in a4 and b4. LBB, left bundle branch; LVAT, left ventricular activation time; PVI, potential to ventricular interval; stim‐LVAT, stimulus to left ventricular activation time

The mean pQRSD and the mean stim‐LVAT was 106.0 ± 12.9 milliseconds and 64.4 ± 13.7 milliseconds respectively. Final pQRS morphology in lead V1 was either a QR or Qr type. In 10 patients, a stimulus to QRS interval was identical under the threshold output with an rSR type morphology presented in V1. The initial r wave diminished with the morphology of QRS developed into QR type and stim‐LVAT remained consistent as the output elevated (Figure 1). In most patients, the dynamic change of stim‐LVAT could be observed during the lead screwing‐in process (Figure 2). When patients were grouped by different LVIDd, larger LVIDd (≥ 55 mm) was documented in 44 patients and small LVIDd (< 55 mm) was documented in 102 patients. Statistic difference of stim‐LVAT (72.7 ± 16.1 ms vs 63.6 ± 8.1 ms, *P* < .0001) existed between these groups.

LBB potentials were observed in 89 patients. The PVI was 18.5 ± 5.6 millisecond and the amplitude of LBB potential was 0.3 ± 0.1 mv. LBBP was successful in 52 of 55 (98.5%) patients with SSS and 43 of these patients presented LBB potentials. In addition, 80 out of 93 patients with AVB were successful in LBBP, while LBB potentials were recorded in 46 of them. In the 34 AVB patients without LBB potential, 30 patients had a ventricular escape rhythm with complete LBBB morphology or had implanted a temporary pacemaker due to an extremely slow escape rate. LBB potentials appeared more frequently in patients with SSS than those with AVB (82.7% vs 57.5%,*P* = .002). However, no significant differences were found between the LBB potential (+) subgroup and LBB potential (−) subgroup (Table 2) in pQRSD (106.05 ± 12.80 millisecond vs 104.57 ± 10.71 millisecond, *P* = .46), stim‐LVAT (65.90 ± 11.04 millisecond vs 67.28 ± 15.27 millisecond, *P* = .06), and capture threshold (0.49 ± 0.25 @ 0.4 millisecond vs 0.46 ± 0.18 @ 0.4 millisecond, *P* = .42).

**Table 2:** Subgroup analysis of electrical characteristics according to LBB potential

	LBB potential (+)	LBB potential (−)	*P* value
N	89	57	…
Threshold (V)	0.49 ± 0.25	0.46 ± 0.18	.42
R‐wave amplitude (mV)	11.98 ± 5.78	10.62 ± 6.8	.08
Impedance (Ω)	672.65 ± 142.47	657.92 ± 159.23	.23
pQRSD (ms)	106.05 ± 12.80	104.57 ± 10.71	.46
Stim‐LVAT (ms)	65.90 ± 11.04	67.28 ± 15.27	.06

Abbreviations: LBB, left bundle branch; pQRSD, paced QRS duration; stim‐LVAT, stimulus to left ventricular activation time.

### 3.3 Pacing characteristics

**Figure 3:**
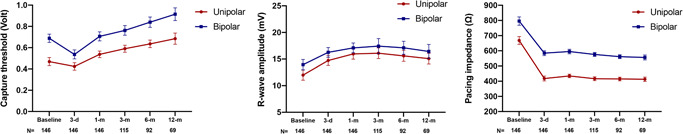
Lead parameters during follow‐up. The pacing impedance decreased significantly at 3 days after operation compared with that during operation. The capture threshold, R‐wave amplitude and impedance remained stable throughout the whole observation period

Unipolar LBBP threshold was 0.48 ± 0.23 V@ 0.4 millisecond while the bipolar threshold was 0.69 ± 0.32 V@ 0.4 millisecond at implantation. The unipolar and bipolar sensed R waves were 11.98 ± 5.62 mV and 13.94 ± 5.59 mV, respectively at implantation. Impedance decreased significantly at 3 days after operation (unipolar 667.96 ± 154.94 Ω vs 418.16 ± 70.75 Ω; bipolar 795.90 ± 156.05 Ω vs 584.52 ± 70.33 Ω, *P* < .001) while the threshold and sensed R‐wave remained stable compared with that at implantation (Figure 3). Lead parameters, including capture threshold, R‐wave amplitude and pacing impedance were stable throughout the whole observation period (ranges from 3 to 18 months), which were showed in Table 3.

**Table 3:** Lead parameters during follow‐up

	Cases	Threshold V@ 0.4 ms		R wave (mv)		Impedance (Ω)	
Time point		Unipolar	Bipolar	Unipolar	Bipolar	Unipolar	Bipolar
During OP	146	0.48 ± 0.23	0.69 ± 0.32	11.98 ± 5.62	13.94 ± 5.59	667.96 ± 154.94	795.9 ± 156.05
3 d	146	0.42 ± 0.16	0.53 ± 0.18	14.73 ± 3.45	16.25 ± 3.29	418.16 ± 70.75*	584.52 ± 70.33*
1 mo	146	0.53 ± 0.15	0.70 ± 0.20	15.59 ± 4.78	16.87 ± 4.08	434.78 ± 57.59	595.13 ± 73.38
3 mo	115	0.59 ± 0.15	0.77 ± 0.20	16.27 ± 4.10	17.60 ± 5.80	418.18 ± 69.60	580.38 ± 69.26
6 mo	92	0.64 ± 0.17	0.84 ± 0.23	15.60 ± 3.66	17.11 ± 4.40	415.24 ± 54.94	559.10 ± 61.07
12 mo	69	0.67 ± 0.19	0.92 ± 0.24	14.90 ± 3.08	16.58 ± 4.17	411.87 ± 56.67	554.97 ± 62.68

Abbreviation: OP, operation.

* The electrode impedance decreased significantly in 3 d postoperation follow‐up comparing with that during operation, *P* < .0001.

### 3.4 Echocardiographic characteristics

**Figure 4:**
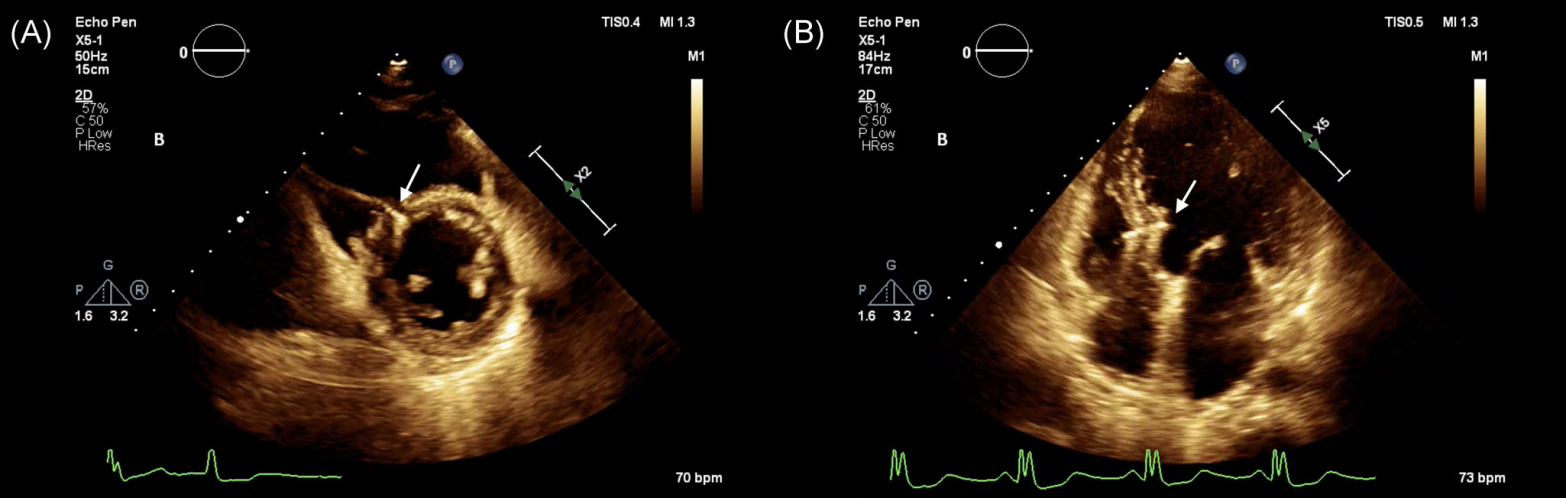
Transventricular septal method for LBBP. A transventricular septal method was applied during the LBBP procedure. Via echo, the tip of active lead was observed to protrude toward the LV cavity while just beneath the endocardium at 3 days after operation. A, Short axis view; B, apical four‐chamber view. LBBP, left bundle branch pacing; LV, left ventricular

**Figure 5:**
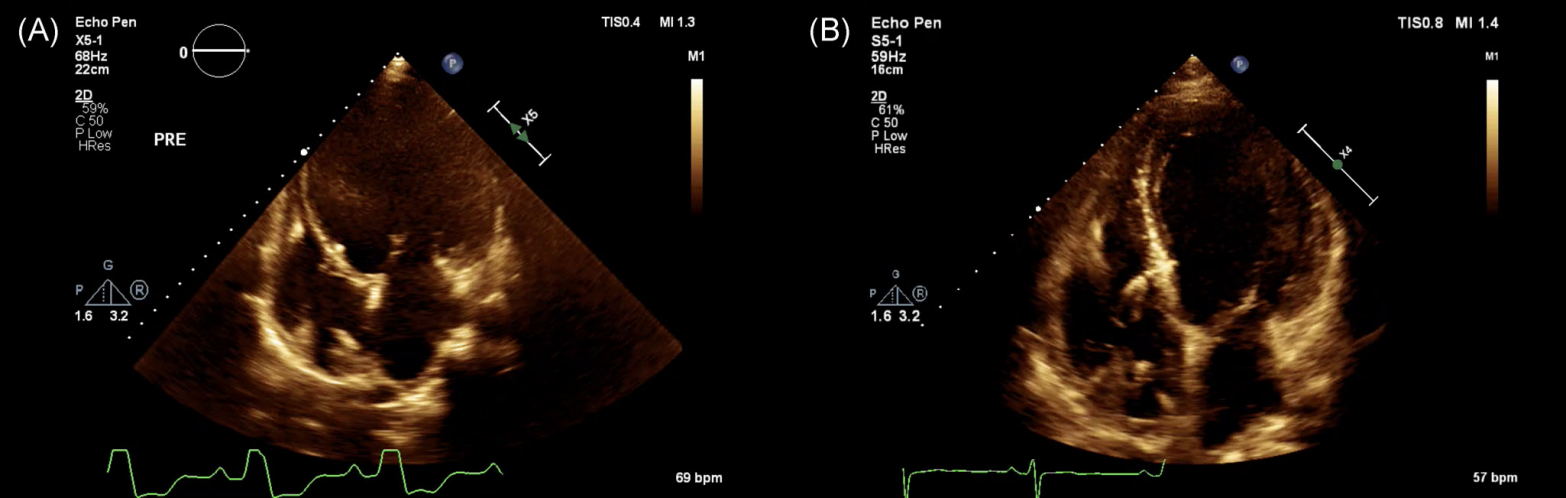
Septum remodeling after LBBP. In a patient complicated with DCM who underwent LBBP for resynchronization therapy, the phenomenon of “lead protruding” toward the LV cavity disappeared after remodeling of the ventricular septum during follow‐ups. The patient's IVSd increased from 7.8 mm to 10.2 mm. A, Three days after operation; B, 1 year after operation. DCM, dilated cardiomyopathy; IVSd, interventricular septal thickness; LBBP, left bundle branch pacing; LV, left ventricular

Postprocedural echocardiograms were performed at 3 days after operation and each follow‐up visit. Typical image of LBBP lead through the interventricular septum with the tip right beneath the left ventricular endocardium was showed by echocardiogram in Figure 4. In three patients complicated with dilated cardiomyopathy (DCM), the tip of active lead was observed to protrude toward the LV cavity while just beneath the endocardium. This “bulging” phenomenon disappeared after remodeling of the ventricular septum during follow‐ups (Figure 5). No significant worsening of tricuspid regurgitation was documented in this cohort.

The echocardiographic data including LVEF (57.5 ± 10.7% vs 56.4 ± 8.4%,*P* = .85), IVSd (9.4 ± 5.4 mm vs 9.9 ± 4.2 mm,*P* = .44), and LVIDd (51.7 ± 5.8 mm vs 53.6 ± 5.6 mm, *P* = .35) remained stable within the 12‐month follow‐up period compared with that at admission. In 14 patients with DCM, LVIDd (64.0 ± 7.5 mm vs 53.1 ± 6.0 mm, *P* < .001) was decreased and LVEF (30.1 ± 5.0%vs 50.6 ± 10.4%, *P* < .001) was increased significantly at 6 months follow‐up visit compared with that at admission.

### 3.5 Safe endpoints

Temporary right bundle branch block (RBBB) occurred in 16 patients during the procedure, 13 patients recovered immediately after the operation, and three patients recovered a day after the operation. About 15 out of 16 temporary RBBB cases occurred when introducing the sheath through the TV ring except for one case occurred during lead screw‐in, in which a barely visible right bundle branch (RBB) potential was observed from the tip electrode of the 3830 lead under unipolar fashion. In the 15 patients mentioned above, vertical displacement of heart was documented in nine patients. Acute perforation of the ventricular septum was documented in three patients, and all of these three patients were female with one patient complicated with DCM. In this patient with DCM, the IVSd was 7.2 mm and a stable myocardial capture threshold was obtained at 1.0 V@ 0.4 mV while the LBB capture threshold was 1.6 V@ 0.4 mv during the lead implantation, at which point we screwed in the lead for two more rounds to achieve a better threshold, and then perforation happened. In the other two patients experienced acute perforation, we were able to record a LBB potential with low amplitude from the tip electrodes, but perforation happened when we were performing one more round of screw‐in to achieve a better potential amplitude. For all these three patients had perforation, a decrement of unipolar impedance to below 500 Ω along with a capture threshold increment was noted during the procedure. The leads were successfully repositioned and no pericardial effusion or cerebral ischemia was observed in any of these patients. Another patient developed a late lead dislodgment and was revised successfully. No patients developed device or lead infections, chronic capture threshold elevation, and aorta or coronary artery injury.

## 4 DISCUSSION

The present study analyzed the feasibility and safety of LBBP via short‐term and intermediate‐term observations of a Chinese cohort. The main findings of this study are as follows: (a) LBBP has a high success rate and satisfied and stable lead parameters during an intermediate‐term observation. (b) There were no serious complications with this procedure. The results provided substantial evidence for optimum design of large randomized controlled trials about this technique.

Many studies have suggested that due to the electrical and ventricular asynchrony in the left ventricle, the high burden of RVA pacing might be detrimental to specific subgroups of patients.^10^, ^11^ Among the various physiological pacing strategies that are available, LBBP has been applauded as a feasible strategy for patients with heart failure, especially for those who failed in traditional BiV or His bundle pacing (HBP).^12^ Initial investigation also confirmed the safety and feasibility of LBBP during short‐term and medium‐term follow‐ups.^7^, ^8^, ^13^, ^14^, ^15^, ^16^ Although a previous study demonstrated that the electric parameters remained stable within a mean follow‐up duration of 3 months,^17^ only few studies have investigated the intermediate‐term outcomes of this new technique named LBBP. In our present study, 164 participants were recruited, and almost half of them had a follow‐up period longer than 1 year. The pacing implantation indications included sick sinus disease, atrioventricular block, and CRT, which were similar to the regular clinical practice.

LBB fans out directly from the branching point of His bundle beneath the membranous septum with well‐defined dimensions.^18^ In light of LBB's anatomy, satisfied lead parameters could be expected.^19^ In this study, we confirmed that capture threshold, impedance, and R‐wave amplitude of LBBP were comparable with that of traditional RVA pacing and remained stable throughout the whole observation period. Considering the feasibility LBBP has demonstrated, it's suitable to promote this technique in clinical practice.

According to Upadhyay et al's^20^ study, left intra‐hisian block is the most common pathophysiological mechanism of LBBB pattern. In that case, a relatively distal site of location may stand more chance in correcting LBBB. There were 16 patients tormented by heart failure complicating with LBBB in this cohort, and 14 out of these 16 patients’ LBBB was corrected by LBBP in lieu of permanent HBP. Though LBBP and HBP have a similar physiological pacing mechanism, that is, the rapid recruitment of left His‐Purkinje system, some drawbacks such as higher capture thresholds, lower R‐wave amplitudes, atrial oversensing, and increased risk for lead revisions are associated with HBP.^21^, ^22^ In the present study, we also proved that LBBP is a preferable option for resynchronization therapy, especially for patients with a failed HBP attempt.

About 11.0% patients failed in the LBBP procedure in this cohort. Most of the patients were complicated with myocardial fibrosis, cardiac hypertrophy, or structural changes, especially large right atrium which might lead to cardiac rotation and insufficient sheath support. In our experience, patients carried the above‐mentioned features might increase the difficulty of LBBP procedure as well as extend the operation time. However, with the improvement of instruments, a higher success rate could be expected in the near future in this population. On the other hand, acute micro lead dislodgement was another vexing problem in the first few patients of our study. We later concluded that reducing the torque of the lead in the sheath before withdrawal of the sheath and reserving adequate lead slack might reduce the possibility of lead dislodgement during or after the procedure.

LVAT represents the local depolarization of the ventricular myocardium beneath the unipolar chest electrodes of V5 or V6^23^ and has been proved to be a suitable subrogate indicator of evaluating left ventricular activation.^7^ It may be deviated by ventricular hypertrophy, ventricular dilation or fibrosis of myocardium. Huang et al^7^ believe that the dynamic changes of stim‐LVAT, such as abruptly shortening with increased output and remaining the shortest and consistent at both low and high outputs, are convincing evidence of LBB capture. Besides, an absolute value of stim‐LVAT may be helpful and practicable in predicting the capture of LBB. In our study, the duration of stim‐LVAT was less than 70 millisecond on average and was mildly longer in patients with the dilated left ventricle. Yet, the suitable value of stim‐LVAT that could be recommended still needed to be investigated in different populations.

Existence of LBB potential during the LBBP procedure can be considered as the strongest evidence of lead being in the periphery of LBB.^24^ The amplitude of LBB potential may be affected by many factors such as the direction of the wavefront, the velocity of conduction, the distance of the bundle branch, and the signal of far‐field or near field.^8^ On the other hand, without retrograde activation of the LBB, the potential may not be visualized in patients with complete LBBB or escape rhythm resulting from a non‐LBB fascicle.^7^ The LBB potential was not recorded in about one‐third patients in our study. No differences lay in pQRSD, stim‐LVAT, and capture threshold between LBB potential (+) and LBB potential (−) subgroups. In that case, we concluded that the existence of LBB potential was not a prerequisite for a successful LBBP procedure.

The right bundle branch is a slender structure 1 to 2 mm in diameter that runs without branching through the interventricular septum and about 1 to 1.5 mm beneath the right interventricular septal endocardium until it reaches the anterolateral papillary muscle of the right ventricle.^25^ Transient injury was unavoidable in some cases during the procedure and would not bring about serious consequences in most occasions. Based on our experience, the following points might be vital to avoid unnecessary RBB injury: (a) keeping the lead in the sheath when introducing the sheath through the TV ring until reach the position that might be appropriate; (b) avoiding screwing in the lead where an RBB potential is recorded from the tip electrode of 3830 lead; (c) preshaping the sheath in patients with cardiac rotation, especially for those with vertical displacement of heart; and (d) avoiding advancing the sheath toward the anterosuperior area of the septum. What's more, before the LBBP procedure, the performance of backup right ventricular pacing for patients with CLBBB was strongly recommended.

Septal perforation and coronary artery injury need to be avoided during the procedure.^12^ In our study, three cases of perforation were documented and no septal branch injury was recorded. According to our experience obtained from this study, (a) elder female patients were more likely to have loose myocardial tissue and therefore develop perforation; (b) a slow and steady lead screwed‐in was necessary during the procedure; (c) the continuous monitoring of unipolar impedance and paced morphology during the procedure was important in avoiding perforation; (d) if perforation does happen, lead reposition is strongly recommended; in light of delayed perforation, pulling the lead back in situ for the sake of good lead parameters should be avoided; and (e) the pursuit of unnecessary high potential amplitude and low threshold might increase the risk for perforation. Lead stability interfered by constant contraction of myocardium and progress of fibrosis due to deep septal injury is another question that needs to be considered. In the current article, LBBP offered stable and reliable electrical parameters over time in all patients. This result also confirmed the safety of LBBP in an intermediate‐term observation.

TVR due to restriction of the leaflet motion by the lead may occur after implantation.^26^ No significant TVR deterioration was found in our study. The cardiac function evaluated by echocardiography remained stable in most of the patients. In the subgroup had resynchronization therapy, both the symptom and echocardiographic data improved. Zhang et al^27^ has evaluated the value of LBBP in resynchronization therapy in a small cohort with LBBB, and their results were consistent with ours in improving the NYHA functional class and echocardiographic index. However, randomized control trails with larger sample size are still needed to verify this effect.

### 4.1 Limitations

This study was a prospective and observational study in a single center. We focused on the safety and feasibility of LBBP in short‐ and intermediate‐term. Further prospective randomized controlled trials are requested to assess the safety and potential beneficial effects of LBBP in a longer term. The electrical and mechanic synchrony and hemodynamic effect of LBBP, especially in the long run, have not been discussed in the present manuscript and should be investigated in further studies.

## 5 CONCLUSION

LBBP is a feasible and safe pacing modality for patients with pacing indications. It has a high success rate, satisfied and stable lead parameters and fewer complications in short‐term and intermediate‐term observations.

## AUTHOR CONTRIBUTIONS

BC conceived and designed this experiment; JG and LL recruited the subjects and collected the clinical data. XH and SC conducted the laboratory testing. FM, MS, XH, SC, QL, and DC helped in data analysis. JG and LL wrote the manuscript. All authors read and approved the final manuscript.

## CONFLICTS OF INTEREST

The authors declare that there are no conflicts of interest.

## ETHICAL APPROVAL

The study was approved by the Ethics Committee of Xiamen Cardiovascular Hospital, Xiamen University and was performed in line with the principles of the Declaration of Helsinki. Informed consent was obtained from all participants.
